# Reduced CA2–CA3 Hippocampal Subfield Volume Is Related to Depression and Normalized by l-DOPA in Newly Diagnosed Parkinson’s Disease

**DOI:** 10.3389/fneur.2017.00084

**Published:** 2017-03-17

**Authors:** Orsolya Györfi, Helga Nagy, Magdolna Bokor, Ahmed A. Moustafa, Ivana Rosenzweig, Oguz Kelemen, Szabolcs Kéri

**Affiliations:** ^1^Department of Neurology, Nyírö Gyula Hospital, National Institute of Psychiatry and Addictions, Budapest, Hungary; ^2^National Institute for Medical Rehabilitation, Budapest, Hungary; ^3^Department of Neurology, Semmelweis University, Budapest, Hungary; ^4^School of Social Sciences and Psychology, Western Sydney University, Sydney, NSW, Australia; ^5^Marcs Institute for Brain and Behavior, Western Sydney University, Sydney, NSW, Australia; ^6^Sleep and Brain Plasticity Centre, Department of Neuroimaging, IOPPN, King’s College and Imperial College London, London, UK; ^7^Sleep Disorders Centre, Guy’s Hospital, Guy’s and St Thomas’ NHS Foundation Trust, London, UK; ^8^Faculty of Medicine, Department of Behavioral Sciences, University of Szeged, Szeged, Hungary; ^9^Department of Cognitive Science, Budapest University of Technology and Economics, Budapest, Hungary; ^10^Faculty of Medicine, Department of Physiology, University of Szeged, Szeged, Hungary

**Keywords:** Parkinson’s disease, hippocampus, depression, l-DOPA, MRI

## Abstract

Hippocampal dysfunctions may play an important role in the non-motor aspects of Parkinson’s disease (PD), including depressive and cognitive symptoms. Fine structural alterations of the hippocampus and their relationship with symptoms and medication effects are unknown in newly diagnosed PD. We measured the volume of hippocampal subfields in 35 drug-naïve, newly diagnosed PD patients without cognitive impairment and 30 matched healthy control individuals. Assessments were performed when the patients did not receive medications and after a 24-week period of l-DOPA treatment. We obtained a T1-weighted 3D magnetization-prepared rapid acquisition gradient echo image at each assessment. FreeSurfer v6.0 was used for image analysis. Results revealed a selectively decreased CA2–CA3 volume in non-medicated PD patients, which was normalized after the 24-week treatment period. Higher depressive symptoms were associated with smaller CA2–CA3 volumes. These results indicate that the CA2–CA3 subfield is structurally affected in the earliest stage of PD in the absence of cognitive impairment. This structural anomaly, normalized by l-DOPA, is related to depressive non-motor symptoms.

## Introduction

Recently, the structure and function of the hippocampal formation received widespread attention in Parkinson’s disease (PD) as a potential neural substrate of cognitive dysfunctions and other non-motor symptoms (e.g., depression, impulse control disorders, and hyposmia) (1). The hippocampal complex forms dense connections with the neocortex and subcortical structures, receives dopaminergic innervation from mesencephalic centers, and participates in learning, memory, multimodal sensory integration, spatial navigation, and emotion regulation (2, 3). From a structural point of view, the hippocampus comprises several histologically distinguishable modules, such as the Cornu Ammonis regions, dentate gyrus (DG), presubiculum, and subiculum, which may be specifically affected in different diseases (2, 3).

Although not without controversy and technical limitations (4), recent advances in *in vivo* hippocampal subfield measurements provide a unique opportunity to gain insight into hidden structural alterations in brain diseases. However, currently, there is no consensus about the most appropriate imaging method and analytical software for such measurements. FreeSurfer, a publicly available software (5), is suitable for the automated segmentation of the hippocampus in large and heterogeneous clinical samples. In several studies, FreeSurfer has been successfully used to find relationships between clinical features and hippocampal structure in various neurological and psychiatric illnesses (6–10).

Although several studies have demonstrated hippocampal atrophy even in non-demented PD patients (11–15), the results are still controversial (16–18). Pereira et al. (19) demonstrated smaller CA2–CA3 and CA4–DG subfields in PD patients relative to matched healthy control individuals, which were linked to less efficient learning performances. In the same sample, reduced subiculum volume was related to visual hallucinations (19). Beyer et al. (20) found a significant association between deficient recall on a verbal memory task and atrophy in CA1, CA3, and subiculum in drug-naïve, newly diagnosed PD patients. Although the study of Beyer et al. (20) reported results from a large sample of newly diagnosed PD patients, matched control subjects were not included, and the effect of medications was not explored (20). Structural abnormalities in the hippocampus may be associated with mild cognitive impairment (MCI) (21) and depression (22) in PD.

An increasing number of studies indicate that dopaminergic medications modulate structural and functional plasticity in the hippocampus, including long-term synaptic depression and potentiation, subunit composition of glutamate receptors, and neurogenesis in the DG (23–25). Despite the fact that these findings are important for acquiring an understanding of non-motor symptoms and the effects of dopaminergic medications in PD, the influence of commonly used antiparkinsonian agents on human hippocampal structure has not been elucidated yet.

Therefore, the current study had the following aims: (a) to explore possible structural differences in hippocampal subfields between drug-naïve, newly diagnosed PD patients without cognitive deficits and matched healthy control subjects; (b) to investigate the relationship between hippocampal structural alterations and clinical characteristics with a special reference to depressive symptoms; and (c) to conduct a longitudinal assessment focusing on the effect of l-DOPA on hippocampal subfield structure.

Our hypotheses were as follows: (a) drug-naïve, newly diagnosed PD patients show reduced CA2–CA3 and CA4–DG volumes (19). (b) Volume reductions are associated with depressive symptoms (22). (c) l-DOPA medication is associated with increased hippocampal subfield volumes (25).

## Materials and Methods

### Participants

Thirty-five patients with PD and 30 healthy subjects matched for age, gender, education, socioeconomic status, IQ, and body mass index were enrolled. The study was conducted at the National Institute of Psychiatry and Addiction, Budapest, Hungary. We contacted eight outpatient centers specialized in the diagnosis and treatment of PD. The clinical diagnosis was made, and the scales were administered by trained neurologists and psychiatrists. All patients meet the UK Parkinson’s Disease Society Brain Bank clinical diagnostic criteria (26).

We used the following scales for the clinical and demographic characterization of the patients: Hoehn–Yahr scale (27) (number of patients in each stage: 1:7, 1.5:5, 2:20, 2.5:3), Unified Parkinson’s Disease Rating Scale (UPDRS) (28), Hamilton Depression Rating Scale (HAM-D), Hamilton Anxiety Rating Scale (HAM-A) (29), Hollingshead Four-Factor Index for socioeconomic status (30), and the Wechsler Adult Intelligence Scale (WAIS-R) for general intellectual functions (31). Impulsive–compulsive spectrum behavior was evaluated according to the criteria of Voon and Fox (32). MCI was excluded based on the criteria of the Movement Disorder Society Task Force guideline (33). Participants received the Montreal Cognitive Assessment, Rey’s Auditory Verbal Learning Test, semantic/phonological fluency, Visual Form Discrimination Test, and the Benton Facial Recognition Test (19, 34). General exclusion criteria included a history of neurological and psychiatric disorders including MCI and impulsive–compulsive symptoms, diabetes mellitus, hypertension, and smoking. The participants did not lose or gain more than 2% of their body weight during the study period.

After the baseline testing in non-medicated state, PD patients started l-DOPA therapy for 24 weeks (mean dose at follow-up: 450.0 mg/day, range: 300–600 mg/day). The selection of the appropriate dose of l-DOPA medication was at the discretion of the treating clinician. After the 24-week period, patients and controls were re-evaluated. The clinical and demographic data are summarized in Tables 1 and 2.

**Table 1:** **Demographic and neuropsychological characteristics of the participants**.

	Parkinson’s patients (*n* = 35)	Control subjects (*n* = 30)
Age (years)	51.9 (7.2)	51.3 (6.4)
Gender (male/female)	21/14	20/10
Education (years)	14.5 (3.8)	14.1 (3.9)
Socioeconomic status	37.7 (9.1)	37.5 (10.6)
BMI (kg/m^2^)	24.7 (7.6)	24.9 (8.1)
IQ	106.8 (11.0)	104.6 (11.0)
Montreal Cognitive Assessment	29.0 (3.2)	28.7 (2.9)
RAVLT—learning	42.8 (7.5)	41.8 (7.9)
RAVLT—recall	9.8 (2.7)	9.4 (2.5)
RAVLT—recognition	15.9 (2.1)	15.7 (1.9)
Semantic fluency	20.6 (4.4)	20.1 (5.0)
Phonological fluency	13.8 (4.2)	14.0 (4.5)
Visual Form Discrimination Test	31.0 (1.6)	31.2 (1.7)
Benton Facial Recognition Test	49.8 (2.7)	49.5 (2.6)

*Data are mean (SD) with the exception of gender distribution. There were no significant differences between Parkinson’s patients and healthy control participants (ps > 0.5)*.

*RAVLT, Rey’s Auditory Verbal Learning Test; BMI, body mass index*.

**Table 2:** **Clinical measures in Parkinson’s patients**.

	Baseline	Follow-up	*t*	*p*
UPDRS total	38.3 (5.1)	33.5 (5.7)	3.46	0.001
UPDRS motor	25.4 (3.8)	20.7 (4.4)	3.95	0.0003
HAM-D	11.6 (7.0)	7.6 (4.0)	2.45	0.02
HAM-A	3.5 (2.7)	4.0 (3.4)	−0.50	0.62

*Data are mean (SD). Results from the baseline, non-medicated condition were compared with those from the follow-up, medicated condition by two-tailed *t*-tests*.

*UPDRS, Unified Parkinson’s Disease Rating Scale; HAM-D, Hamilton Depression Rating Scale; HAM-A, Hamilton Anxiety Rating Scale*.

### Structural Magnetic Resonance Imaging

During the acquisition and processing of images, we followed the protocol of Marizzoni et al. (35), which provided evidence for the longitudinal reproducibility of automated hippocampal subfield measurements (35). The protocol included a structural T1 volume at each assessment [Philips Achieva 3 T scanner, magnetization-prepared rapid acquisition gradient echo, 3D sagittal acquisition, square field of view = 256 mm, acquisition matrix: 256 × 256, voxel size: 1 mm × 1 mm × 1 mm, TI = 900 ms, TE (shortest) = 3.16 ms, flip angle: 9 degrees, no fat suppression, full *k* space, no averages, acquisition time: 6 min and 50 s, acceleration factor: 2].

For image processing, we used the neuGRID platform and the longitudinal pipeline of FreeSurfer v6.0 with the “hipposubfields” flag^1^ (36). After a within-session averaging of T1-weighted images, we performed an automatic hippocampal subfield segmentation (5). Image processing included the following steps: (1) correction for within-subject head motion; (2) removal of non-brain tissue using a hybrid watershed/surface deformation algorithm; (3) affine registration to Talairach space; and (4) segmentation of cortical and subcortical structures with a Probabilistic Brain Atlas (37).

The FreeSurfer module for hippocampal segmentation is based on a Bayesian model with Markov random field priors (5). In FreeSurfer v6.0, a newly developed version of the hippocampal segmentation tool has been implemented (38). Briefly, ultra-high-resolution (0.13 mm) *ex vivo* MRI scans from 15 autopsy samples were manually segmented, together with a delineation of neighboring structures from *in vivo*, T1-weighted images (1 mm resolution). The manual labels from the *ex vivo* and *in vivo* scans were integrated to establish an atlas of the hippocampal formation with a new Bayesian inference algorithm to detect local variations in MRI contrast. This new method has several advantages over previous versions of the FreeSurfer hippocampal segmentation module (38, 39).

**Figure 1:**
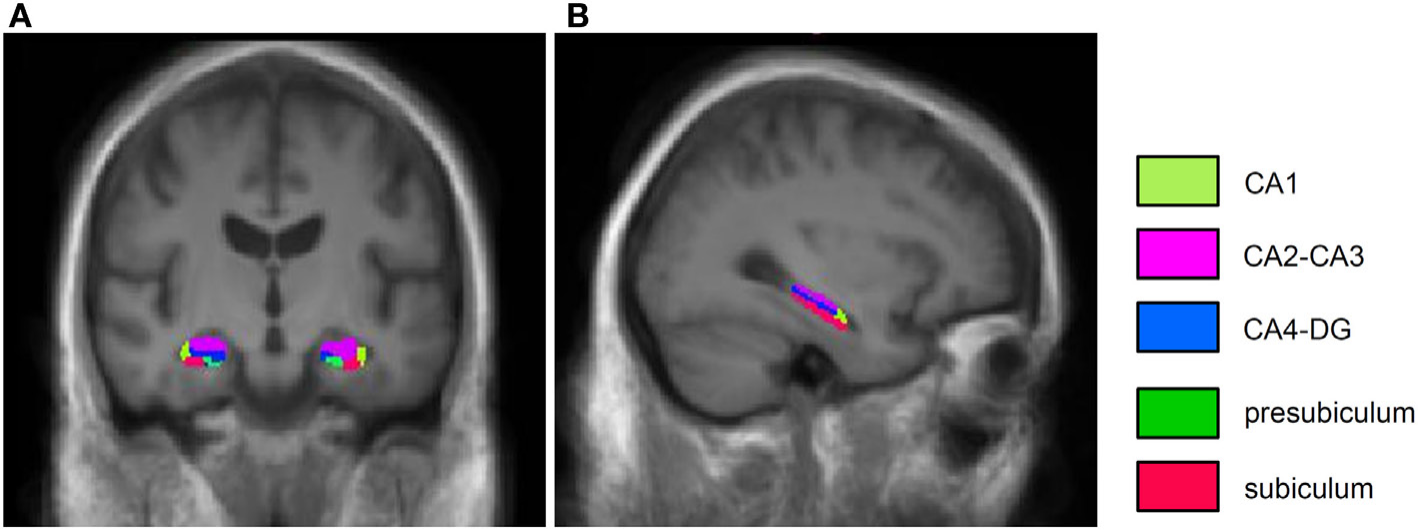
**Coronal (A) and sagittal (B) T1-weighted images from the average output of FreeSurfer hippocampal segmentation from healthy individuals**.

We segmented and measured the volume of the following subfields: CA1, CA2–CA3, CA4–DG, subiculum, and presubiculum (Figure 1). Hippocampal subfield volumes were adjusted by the total intracranial volume (40). The fimbria and the hippocampal fissure were not included in the analysis because the segmentation of these small structures is inaccurate, and their longitudinal reproducibility is insufficient (9, 35). We averaged the volumetric data across hemispheres because we did not establish a hypothesis regarding laterality (volumetric differences between the left and right hippocampus), and our exploratory analyses did not indicate significant differences between left and right hippocampal subfields in PD. In this way, we could reduce the number of variables in our relatively small sample.

In 15 healthy control individuals, we validated the FreeSurfer hippocampal subfield measurement against the widely used AdaBoost machine-learning segmentation method (20, 41, 42). AdaBoost employs statistical rules for subfield segmentation based on numerous voxel-specific features (e.g., image gradients, local curvatures, classification of gray or white matter, and stereotaxic position of the hippocampus) using a training dataset. The AdaBoost algorithm labels each voxel in MRI images to be segmented and delineates hippocampal subfields. AdaBoost is able to detect hippocampal atrophy at least as effectively as manual segmentation and FreeSurfer (43). Intraclass correlation coefficients (ICCs) between AdaBoost and FreeSurfer delineations were calculated using a two-way random ANOVA model with absolute agreement (44). We found good to excellent ICCs according to the definition of Cicchetti (45) (CA1: 0.68; CA2–CA3: 0.76; CA4–DG: 0.79; subiculum: 0.67; presubiculum: 0.74).

Finally, we evaluated the test–retest reliability of automated FreeSurfer measures by calculating Pearson’s correlation coefficients between subfield values measured at baseline and follow-up. We found high correlation coefficients indicating good test–retest reliabilities (CA1: 0.82; CA2–CA3: 0.81; CA4–DG: 0.80; subiculum: 0.84; presubiculum: 0.84).

### Voxel-Based Morphometry (VBM)

A whole-brain VBM was performed to detect possible gray matter differences between PD patients and controls. We used the VBM8 toolbox of SPM8^2^ and the Diffeomorphic Anatomical Registration Through Exponentiated Lie Algebra toolbox under a MATLAB 7.14 platform (MathWorks, Natick, MA, USA) (46, 47). Image analysis involved the following steps: (1) segmentation of the raw MRI images in native space into gray matter, white matter, and cerebrospinal fluid; (2) normalization of images to gray matter and white matter templates in stereotactic space; (3) automatic segmentation of normalized images; and (4) smoothing (8-mm, full-width, half-maximum Gaussian kernel).

We applied general linear model for the statistical analysis of VBM data (voxel-wise estimation of the local amount of gray matter) with total gray matter volume as a covariate. The voxel-wise threshold was *p* < 0.001, uncorrected (extent threshold: *K* = 20 voxels). The extent threshold was determined with AlphaSim employing Monte Carlo simulations (number of iterations: 1,000, alpha-level: 0.05) (48).

### Statistical Analysis of Hippocampal Subfields and Clinical Measures

We used STATISTICA 12 software (StatSoft, Tulsa) for data analysis. Kolmogorov–Smirnov tests and Levene’s tests did not indicate significant deviations from normal distribution and inhomogeneity of variance, respectively (*p*s > 0.5). Therefore, we performed an ANOVA on hippocampal subfield volumes. The between-subjects factor was the experimental group (PD patients vs. control individuals). The within-subject factors were assessment session (baseline, non-medicated state in PD vs. follow-up, PD patients on l-DOPA) and hippocampal subfields. Tukey honestly significant difference (HSD) tests for unequal samples were used for *post hoc* comparisons. Cohen’s effects size values (*d*) were also calculated. Demographic data were compared with two-tailed Student’s *t*-tests. Pearson’s product moment partial correlations were calculated between scales assessing depression (HAM-D), anxiety (HAM-A), PD symptoms (UPDRS total and motor subscales), and hippocampal subfield volumes. Fisher *r*-to-*z* transformation was used to compare correlation coefficients. The level of statistical significance was set at alpha <0.05. We used the false discovery rate (FDR) method for the correction of multiple comparisons.

## Results

### Hippocampal Subfields

Figure 1 depicts the hippocampal segmentation results. Hippocampal subfield volumes are shown in Table 3. Analysis of variance (ANOVA) indicated a significant difference between PD patients and control subjects (a main effect of experimental group) [*F*(1,63) = 4.01, *p* < 0.05, η^2^ = 0.06]. Furthermore, there was a significant main effect of assessment session (baseline vs. follow-up) [*F*(1,43) = 30.54, *p* < 0.001, η^2^ = 0.33] and hippocampal subfields [*F*(4,252) = 384.51, *p* < 0.001, η^2^ = 0.86]. We found two-way interactions between group and assessment session [*F*(1,63) = 15.91, *p* < 0.001, η^2^ = 0.20], and assessment session and hippocampal subfields [*F*(4,252) = 15.69, *p* < 0.001, η^2^ = 0.20]. Most importantly, there was a three-way interaction among experimental group, assessment session, and hippocampal subfields [*F*(4,252) = 13.59, *p* < 0.001, η^2^ = 0.18].

**Table 3:** **Hippocampal subfield volumes (mm^3^)**.

Subfields	Parkinson’s disease (*n* = 35)			Control subjects (*n* = 30)			Effect size
	Mean	SD	95% CI	Mean	SD	95% CI	*d*
**Baseline**							
CA1	342.3	69.3	318.5–366.1	350.6	70.8	324.1–377.0	0.12
CA2–CA3*	742.3	97.9	708.7–776.0	831.6	88.6	798.5–864.7	0.87
CA4–DG	543.5	73.5	518.2–568.7	567.8	66.1	543.2–592.5	0.34
Subiculum	589.3	75.2	563.5–615.1	574.6	90.9	540.7–608.6	0.18
Presubiculum	388.5	87.8	358.3–418.7	415.2	77.8	386.2–444.3	0.32
**Follow-up**							
CA1	345.0	71.6	320.4–369.6	355.6	73.0	328.4–382.9	0.15
CA2–CA3	851.7	83.4	823.1–880.3	838.6	88.0	805.7–871.5	0.15
CA4–DG	564.3	80.8	536.5–592.0	569.3	60.2	546.8–591.8	0.06
Subiculum	572.8	76.0	546.7–599.0	576.2	98.1	539.6–612.9	0.05
Presubiculum	386.0	91.0	354.8–417.3	418.5	79.6	388.7–448.2	0.38

At baseline, Parkinson’s patients did not receive medications. Follow-up measurements were conducted after 24 weeks of l-DOPA treatment in the patient group. Healthy control subjects did not receive any medications. Hippocampal subfield volumes (mm^3^) from the patients and control subjects were compared with analysis of variance and Tukey honestly significant difference tests (*p < 0.0001). Effect size values (Cohen’s *d*) were also calculated.

*95% CI, 95% confidence interval; CA, Cornu Ammonis; DG, dentate gyrus*.

Tukey HSD tests conducted on the three-way interaction indicated that the baseline CA2–CA3 volumes were significantly smaller in non-medicated PD patients as compared to the control group (*p* < 0.0001). No between-group differences were found for other hippocampal subfields in the baseline condition (*p*s > 0.7) (Table 3). At the follow-up assessment, we did not find significant differences between PD patients and healthy controls (*p*s > 0.7) (Table 3).

In patients with PD, we observed a significant increase in CA2–CA3 volumes during the follow-up period (non-medicated vs. medicated state, *p* < 0.001). There were no significant changes in the remaining hippocampal subfields (*p* > 0.5).

### Correlations between Hippocampal Subfield Volumes and Clinical Symptoms

**Figure 2:**
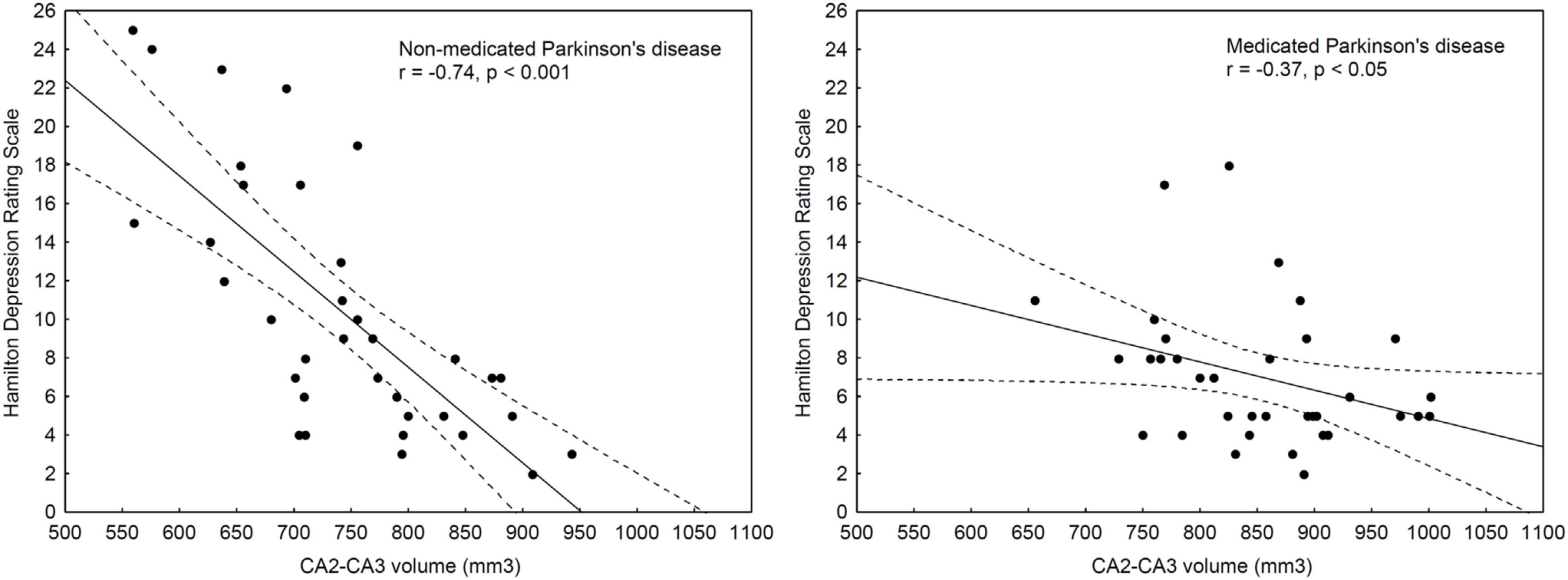
**Correlations between depressive symptoms and CA2–CA3 volumes before (non-medicated, left panel) and after l-DOPA medication (medicated, right panel)**.

We calculated partial correlations, corrected for age, gender, and education, between hippocampal subfields and clinical symptoms (UPDRS total and motor symptoms, HAM-D, and HAM-A). At both baseline and follow-up assessments, more severe depressive symptoms (HAM-D) were associated with smaller CA2–CA3 volumes (*r*_baseline_ = −0.74 and *r*_follow-up_ = −0.37, *p*s < 0.05) (Figure 2), although the correlation coefficient at follow-up was significantly smaller than that at baseline (*Z* = −2.25, *p* = 0.02).

A similar relationship was observed for anxiety, but it was significant only at the baseline assessment (*r*_baseline_ = −0.47, *p* < 0.05). There were no significant correlations for other hippocampal subfields and UPDRS/HAM-D/HAM-A scores (−0.3 < *r*s < 0.3, *p*s > 0.05). When FDR corrections were used, only the relationship between baseline depressive symptoms and CA2–CA3 volume retained significance.

### Voxel-Based Morphometry

There was no significant difference in gray matter volume between PD patients and control subjects even at the screening threshold (*p* < 0.001, uncorrected).

## Discussion

The findings of the present study indicate that the CA2–CA3 subfield of the hippocampal formation is significantly reduced even in the earliest stage of clinically diagnosed PD. Moreover, in the initial period of l-DOPA treatment, this volumetric alteration, associated with depressive symptoms, shows improvement.

The results are in accordance with neuropathological studies indicating a pronounced neurodegeneration in the CA2 field in PD (49), although we were only able to measure a collapsed CA2–CA3 field. Pereira et al. (19) also demonstrated a decreased volume in CA2–CA3, but in their study, a smaller CA4–DG volume was also observed in PD patients. We did not detect significant atrophy in CA4–DG, probably because we investigated newly diagnosed, early-stage PD patients.

The CA2–CA3 subfield is critical in the formation of new memories (50, 51). Moreover, CA2 may be a pivotal interface between brain regions responsible for emotional processing and higher level cognitive functions (52). Considering its role in social memory, unique cytoarchitectonic properties, and neuromodulation, CA2 may be a key target for the treatment of social and emotional dysfunctions in various neuropsychiatric illnesses (52). In line with these findings, we demonstrated that the CA2–CA3 region may be critical in the emergence of early affective symptoms in PD: at baseline, more severe depressive symptoms were associated with smaller CA2–CA3 volumes. At the second assessment, when PD patients received l-DOPA, depressive symptoms were improved, and their correlation with the CA2–CA3 volume was less pronounced. This observation provides insight into the neuronal correlates of the early antidepressive effects of l-DOPA (53). This finding is clinically relevant because a significant proportion of PD patients suffer from depression (pooled prevalence: 22.9%) (54), which markedly affects quality of life (55).

It is worth noting that several studies revealed a widespread hippocampal volume loss in major depressive disorder instead of a circumscribed deficit in certain subfields (3). A possible explanation may be that in our PD patients, depressive symptoms were not as severe as in individuals with a clinical diagnosis of major depressive disorder, or depressive symptoms have distinct mechanisms in major depressive disorder and PD. In patients with PD and comorbid depression, several studies have demonstrated widespread structural alterations in prefrontal and limbic regions, although some research groups failed to detect significant differences between depressed and non-depressed PD patients (56). Others emphasized the special role of the limbic thalamus (57). van Mierlo et al. (22) demonstrated a complex relationship between depression and brain structure in PD: depression scores negatively correlated with bilateral hippocampus and right amygdala volume and positively with anterior cingulate cortex volume. It has been suggested that depression and neurodegenerative processes may share several pathophysiological features, including decreased production of neurotrophic factors, reduced neurogenesis, abnormal synaptic plasticity, and enhanced neuroinflammation in the hippocampus (58).

We found that a 24-week period of l-DOPA treatment restored CA2–CA3 volume in PD patients. This suggests that during the initial stage of PD, dopaminergic medications ameliorate hippocampal structural changes. The mechanism of this effect is not exactly understood. Chiu et al. (25) demonstrated that l-DOPA is able to restore neurogenesis in the DG of mice with bilateral intra-nigral 6-hydroxy-dopamine lesion, together with the improvement of Parkinson-like non-motor behavior. In addition to specific influences on neurogenesis, dopamine is able to modify the synthesis of neurotrophic factors exerting effects on neuronal structure and function (59) and modulating inflammatory responses (60). α-Synuclein triggered microglial activation, and neuroinflammation may be especially relevant in the hippocampus of PD patients (61).

It is essential to consider that non-specific factors, such as overall body weight, nutritional status, local water content, and astroglia activation, may account for changes in brain volume (62–64). To tackle these issues, we controlled body weight and asked the participants not to change their dietary habits and physical activity during the study. Moreover, it is not likely that non-specific factors may selectively affect the CA2–CA3 region.

We also conducted a whole-brain VBM to elucidate a possible extra-hippocampal volume loss. We did not find any evidence for extra-hippocampal structural alterations in newly diagnosed PD patients with spared cognitive functions relative to control participants, which is consistent with previous findings (65, 66). Two recent meta-analyses of VBM studies in PD failed to prove hippocampal gray matter reduction (67, 68), whereas a marked medial temporal lobe atrophy was reported in PD patients with dementia (69). The fact that VBM did not reveal hippocampal volume loss in our patients is neither surprising nor contradictory. Whole-brain VBM without region of interest analysis using hippocampal masks fails to detect even larger hippocampal atrophy than that found in our study (70).

Our study is not without limitations. First, the validity of FreeSurfer hippocampal segmentation has been questioned (4). It seems that the discrepancy between FreeSurfer and manual methods is the largest for CA1 (71, 72). Specifically, by comparing manual segmentation and FreeSurfer, de Flores et al. (71) reported low to moderate ICCs (0.31–0.74) for subiculum, other subfields, and whole hippocampus, but for CA1, the correlation was very small (0.02). The CA2–CA3 and CA4–DG subfields could not be discriminated during manual segmentation, and therefore, the authors reported these subfields together. They concluded that “the correlations between FreeSurfer and manual measurements were reasonable for the SUB (subiculum) and CA2–3-4-DG subfields pooled together” (p. 472) (71).

Manual segmentation and FreeSurfer can similarly detect volumetric differences in CA2–CA3 in clinical and healthy populations (72). The advantage of FreeSurfer is that it allows replicability across different scanners, analytical softwares, and experimental samples in longitudinal studies (35). Another advantage is that FreeSurfer does not require high-resolution T2-weighted scans with hippocampal focus, which is sensitive for motion and other types of MRI artifacts (72). Finally, in a group of healthy individuals, we found convincing correlations between FreeSurfer measures and data obtained from the AdaBoost machine-learning segmentation protocol, which is frequently used in clinical populations (20).

The second limitation is that our study did not include a clinical group receiving placebo. We have no information about spontaneous changes in hippocampal structure during the early course of PD. However, it is unlikely that the CA2–CA3 region might undergo a spontaneous volume expansion. A truly critical question for future studies is when and why medication-related volume compensation is lost and it eventually turns into progressive atrophy.

The third main limitation is that the sample size was small. However, the obtained significance level for CA2–CA3 was convincing with a Cohen’s effect size larger than 0.8 (Table 3). The critical three-way interaction in the ANOVA was also highly significant with a large effect size according to Cohen’s criteria (0.18) (73). It is important to note that it is difficult to recruit *de novo*, non-medicated PD patients because general practitioners and other specialists often start various medications (e.g., antidepressants) before the final diagnosis of PD.

In conclusion, newly diagnosed PD patients exhibit a selective reduction of the CA2–CA3 subfield of the hippocampus, which is ameliorated by l-DOPA during the initial phase of the disease. These findings should be interpreted taking into account the small sample size and the lack of a placebo group. Future studies should assess the long-term effect of dopaminergic medications in relation to progressive brain volume loss and non-motor symptoms of PD.

## Ethics Statement

The study was done in accordance with the Declaration of Helsinki. All participants gave written informed consent. The study was approved by the Hungarian Scientific and Research Committee of the Medical Research Council, Budapest, Hungary. No vulnerable populations were included in the study.

## Author Contributions

OG, HN, MB, OK, AM, IR, and SK designed the study. OG, HN, and SK collected and analyzed the data. IR and AM completed the research design and reviewed the analysis. OG and SK wrote the first draft of the paper, which was reviewed and approved by all authors.

## Conflict of Interest Statement

The authors declare that the research was conducted in the absence of any commercial or financial relationships that could be construed as a potential conflict of interest. The reviewer GM and handling editor declared their shared affiliation, and the handling editor states that the process nevertheless met the standards of a fair and objective review.

## Footnotes

^1^http://freesurfer.net.

^2^http://www.fil.ion.ucl.ac.uk/spm.
